# Preliminary results of pravastatin on non-hemorrhagic vertebral artery dissection: an exploratory randomized and controlled study

**DOI:** 10.1186/s12883-025-04257-7

**Published:** 2025-06-03

**Authors:** Hyun Jin Han, Keun Young Park, Chang Ki Jang, Joonho Chung, Yong Bae Kim, Sung Ho Lee, Kangmin Kim, Hyun-Seung Kang, Jeong Eun Kim, Won-Sang Cho

**Affiliations:** 1 Departments of Neurosurgery, Severance Hospital, Seoul, Republic of Korea; 2 Yongin Severance Hospital, Yongin, Republic of Korea; 3 Gangnam Severance Hospital, Seoul, Republic of Korea; 4 Yonsei University College of Medicine, Seoul, Republic of Korea; 5 Department of Neurosurgery, Seoul National University Hospital, Seoul National University College of Medicine, Seoul, Republic of Korea

**Keywords:** Pravastatin, Pleiotropic effects, Vertebral artery dissection, Magnetic resonance imaging, Pilot study

## Abstract

**Background:**

There is limited information about the pleiotropic effects of statin on cerebrovascular diseases. The authors aimed to investigate the effect of pravastatin on non-hemorrhagic vertebral artery dissection (VAD) in a clinical setting.

**Methods:**

An exploratory randomized and controlled study was designed for the non-hemorrhage VAD (CRIS, KCT00035970). Primary outcomes were 1- and 6-month radiologic changes on vessel wall magnetic resonance imaging (VW-MRI), with secondary outcomes related to the clinical and laboratory parameters, and safety outcomes of the pravastatin use.

**Results:**

Finally, 23 patients were enrolled, consisting of 12 in the pravastatin group and 11 in the control with similar baseline characteristics except the age (55.0 versus 45.5 years, *P* = .01). Morphologic changes in the early period (0–1 month) were more improved or resolved in the pravastatin group with a borderline significance (crude odds ratio: 7.500 [95% confidence interval: 0.921-64.047], *P* = .06). However, age-adjusted analysis showed no difference in morphologic changes between the groups (adjusted odds ratio: 0.853 [95% confidence interval: 0.033-22.020], *P* = .92). The serum level of high-sensitivity C-reactive protein (hs-CRP) in the pravastatin group showed a descending tendency in the early period (Z-score: -1.843, *P* = .07), whereas it significantly increased in the control (Z-score: -2.371, *P* = .02). There were no safety issues at all.

**Conclusion:**

Pravastatin had a borderline significant effect on the recovery of non-hemorrhage VAD with no additional adverse effects. A further study with a larger scale is expected to make more concrete evidences about the pleiotropic effects of statin on the non-hemorrhagic VAD.

**Trial registration:**

The present study registered in a clinical research information service approved by World Health Organization (CRIS, KCT00035970, Registered on 7 March 2019; available at: https://cris.nih.go.kr/cris/search/detailSearch.do?seq=25234%26status=5%26seq_group=12949%26search_page=M).

**Supplementary Information:**

The online version contains supplementary material available at 10.1186/s12883-025-04257-7.

## Introduction

Vertebral artery dissection (VAD) is a pathologic condition characterized by intimal tear and progressive disruption of arterial layer in the vertebral artery. Its annual incidence is reported 1-1.5 per 100,000 persons and it is known as one of major causes of the acute ischemic stroke in young-aged population, accounting up to 25% [1, 2]. With the introduction of vascular imaging modalities, the incidence has increased over 4 times during the past two decades [3]. In a pathophysiologic aspect, intimal tear and subsequent thrombus formation induces the direct parent artery steno-occlusion and embolic events. Moreover, propagation breaks a vessel resilience, resulting in a morphologic change.

While hemorrhagic VAD is fatal and has a high risk of rebleeding, the overall clinical course of non-hemorrhagic VAD is relatively benign [4, 5]. So, most cases of non-hemorrhagic VAD do not require surgical and endovascular treatments. Instead, antithrombotic medication is usually recommended for at least 3 months because of the risk of cerebral infarction up to 13.3% [6–8]. However, there is still no attempt of the medical treatment to alter the disease course, such as endothelial restoration of the injured vessel wall.

Statins are widely used as a mainstay lipid-lowering agent for secondary prevention of atherosclerosis-origin cerebral infarction [9]. Early outcome of the patients with ischemic stroke was better in the presence of statin therapy [10, 11]. Moreover, intensive statin therapy leads to enhancing the stabilization and regression of the atherosclerotic plaque [12–14]. Independent with the low-density lipoprotein cholesterol (LDL-C) lowering effect, clinical and laboratory studies have also supported the pleiotropic effects of statins. Especially, modification of inflammatory response, improvement of endothelial cell function, activation of endothelial progenitor cells is suggested as a few potential mechanisms of statins for the enhancement of endothelial restoration [15–20]. 

Therefore, the authors designed an exploratory randomized and controlled study in order to find radiologic and laboratory clues to the potential effect of pravastatin on the non-hemorrhagic VAD.

## Methods

### Patient selection

The present study was initially designed to enroll a total of 34 patients, consisting of 17 in the pravastatin group and 17 in the control group, with a competitive recruitment in the two tertiary hospitals. From October 2018 to August 2022, the patients were recruited according to the following inclusion criteria: (1) patients over 20 years and under 75 years; (2) radiologically confirmed VAD which was not presented with intracranial hemorrhage and located in the V4 segment (intradural portion); (3) clinical symptoms and signs which developed within 1 month; and (4) clinical symptoms and signs, such as posterior neck pain, occipital headache, dizziness and mild neurological deficits (National Institutes of Health Stroke Scale < 5) which were no need of additional treatments other than medical management. Exclusion criteria were as follows: (1) VAD presenting with bleeding and moderate to severe cerebral infarction; (2) the patients who have already taken the statin for hyperlipidemia and cardiovascular diseases; and (3) the patients with the previous history of adverse events related to the statin and aspirin (Supplementary method S1). The target period for the enrollment was initially 3 years. However, the authors could not help stopping this study in March 2023 in spite of a few extension of the enrolling period, because of the Coronavirus Disease 2019 (COVID-19) pandemic and resultant failure to enroll a target number of the patients. Finally, 12 patients in the pravastatin group and 11 in the control group were enrolled.

### Overview of study protocol

When the patients met the eligibility criteria with informed consents, vessel wall magnetic resonance imaging (VW-MRI), and clinical and laboratory examinations were conducted for the screening. The investigators thoroughly examined the medical record as well as the radiologic and laboratory data, and made a decision for the registration. After the registration, A third party unrelated to the study randomly allocated each patient using a free software, Random Allocation Software 1.0 (available at: https://mahmoodsaghaei.tripod.com/Softwares/randalloc.html).

**Fig. 1:**
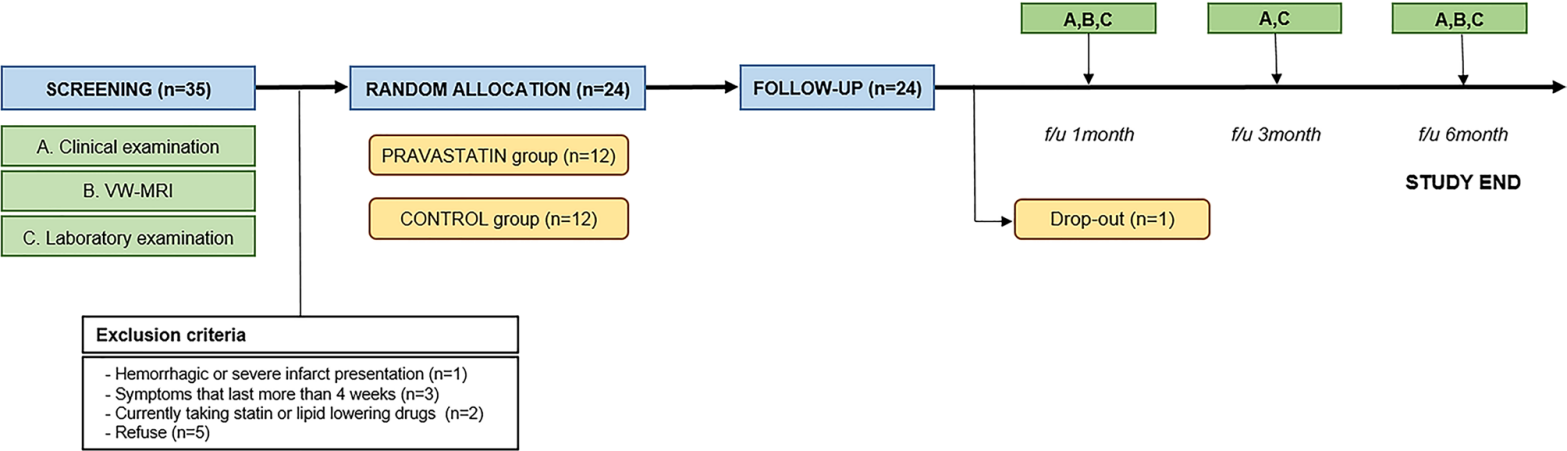
Schematic illustration of the study protocol. VW-MRI, Vessel wall magnetic resonance imaging

The control group started the medications of aspirin 100 mg and antacid. Meanwhile, the pravastatin group began to take a pravastatin 40 mg with aspirin 100 mg and antacid. The patients visited the out-patient clinics in 1, 3 and 6 months after the initial enrollment, and underwent periodic clinical, radiologic and laboratory examinations according to the study protocols. Schematic illustration of the study protocol is illustrated in Fig. 1.

### Primary outcomes

The primary outcomes were evaluated based on the VW-MRI findings checked 3 times at the initial, and in 1 and 6 months from the initial. VW-MRI was performed using a 3.0T MR system (Ingenia CX or Discovery MR750w), which included the imaging sequences of pre- and post-contrast black-blood, proton density and time-of-flight. Details of imaging parameters was described in Supplementary Method S2. Radiologic changes in the early period were evaluated, compared to the initial and 1-month VW-MRIs. Those in the late period were evaluated in comparison of the initial and 6-month VW-MRIs. A senior neuro-interventionist and neurosurgeon independently reviewed and documented the image findings with masking to treatment allocation.

Characteristic MRI findings of the non-hemorrhagic VAD included intimal flap, double lumen sign, intramural hematoma, contrast enhancement of the vessel wall and morphologic changes of steno-occlusion, dilatation and pearl-and string sign. The dissected segment was defined as the area where radiologic features were identified. The curvilinear hyperintensity on T2-weighted imaging, which distinguished the true lumen from the false lumen, indicated the intimal flap. When eccentric artery wall thickening with blood signal features (T1- and T2-weighted images with time taken into account) was observed, an intramural hematoma was reported [21, 22]. The authors measured the diameters of proximal and dissecting segments in each vessel, and calculated the ratio defined as the diameter of involved segment to one of proximal normal segment of a vessel using 3D slicer [23]. The pearl-and-string sign was defined as a focal constriction of the dissected artery segment with a distal site of dilatation.

Of these, intimal flap and double lumen sign were noted as nominal variables. Morphologic change, intramural hematoma and vessel wall enhancement were recorded as 4 categories: aggravated, stationary, improved and resolved. For statistical analysis, the categorized results were dichotomized as “aggravated or stationary” and “improved or resolved”. The case where the aforementioned diameter ratio change was less than 25% was defined as a stationary status. For a qualified assessment of intramural hematoma and vessel wall enhancement, volume and extent of the lesion were measured by a semi-automated segmentation, and each rater independently judged with reference to the previous matched images.

### Secondary outcomes

The secondary outcomes included some laboratory tests such as the high sensitivity C-reactive protein (hs-CRP) and LDL-C, and clinical symptoms and signs. LDL-C level was extracted for evaluating the intrinsic effect of pravastatin. The authors set the statin responder reference as LDL-C reduction of 30% according to the 2013 American College of Cardiology/American Heart Association guideline [24]. For evaluation of anti-inflammatory effects as one of pleiotropic effects of the statin, we measured the level of hs-CRP, which was presented as a sensitive inflammatory marker in patients with cardiovascular disease [25]. Its measurement was conducted using the Turbidimmuno Assay (TBA-200FR, Toshiba Medical Systems) with detection limitation of as low as 0.175 mg/L [26]. 

Of the VAD-related clinical symptoms and signs, headache was assessed using numeric pain rating scale and strictly defined following previous reports. Briefly, the topography of VAD-related headache had the characteristics of acute onset, moderate to severe intensity, persistency lasting longer than 24 h, and initially shock-like and persistently throbbing patterns [27, 28]. Neurologic deficits associated with acute ischemic stroke was initially assessed in a National Institutes of Health Stroke Scale. Clinical examination was performed at every serial follow-up and the findings was documented using the same scale.

### Safety outcomes

The present study evaluated all types of adverse events such as hepatotoxicity, renal toxicity and myopathy related to statin usage, as previously described in the prospective pravastatin pooling project and aspirin-related studies [29, 30]. So, the laboratory examination included total bilirubin, alkaline phosphatase, aspartate transaminase, alanine transferase, creatinine kinase, lactate dehydrogenase, creatinine and blood urea nitrogen. Serum glucose and hemoglobin A1c were checked for monitoring the glucose intolerance and the development of diabetes mellitus as a result of statin usage [31]. In addition, thorough clinical examination was followed up at every visit, including relevant questionnaire for each clinical symptoms and signs.

### Statistical analysis

As there has been no previous report about the use of statin for the patients with non-hemorrhagic VAD, sample size was determined, based on the average number of patients during the last 3 years and the sample size in a previous pravastatin study about its pleiotropic effects for symptomatic carotid stenosis [16]. Taken together, the authors planned to enroll 17 patients in each group with assumption of a drop out of 10%.

Continuous variables were presented as median and interquartile range (IQR), and categorical ones were presented as number and percentage. For the group comparisons of continuous variables, the Mann-Whitney U-test was used. Categorical variables were compared using the Chi-square and Fisher’s exact tests. Crude and age-adjusted odds ratio was calculated to compare between the aggravated/stationary and improved/resolved groups. The changes of hs-CRP and LDL-C levels in the early and late periods were assessed by Wilcoxon signed-rank test. Significance was set at a *P* < .05. Statistical analyses were conducted using IBM SPSS Statistics version 26.0 for Windows (IBM Corp.) and R package version 4.2.2 (R Project for Statistical Computing).

## Results

### Baseline characteristics

Baseline characteristics are described in Table 1. Median age in the pravastatin group was significantly younger than that in the control (45.5 versus 55 years, *P* = .01). Twelve (52.2%) of 23 patients were male. Most of the patients had occipital pain (95.7%) and only one patient in the pravastatin group had a hemiparesthesia as a neurologic deficit. In the radiologic evaluation with VW-MRI, stenotic-occlusive change was the most common initial morphologic finding (52.2%), followed by dilatation (17.4%) and pearl and string signs (13.0%). Intramural hematoma was confirmed in over 90% of the patients in both groups and wall enhancement was detected in all. Between the two groups, there were no significant differences in the clinical symptoms and signs, and VW-MRI findings.

**Table 1:** Baseline characteristics

	Total	Pravastatin Group	Control Group	*P* value
Number of patients	23	12	11	
Age, median (IQR)	47 (38–68)	45.5 (38–57)	55 (42–68)	**0.01**
M: F	12:11	7:5	5:6	0.54
Current smoker, n (%)	5 (21.7)	2 (16.7)	3 (27.3)	0.64
Hypertension, n (%)	8 (34.8)	4 (33.3)	4 (36.4)	1.00
Clinical presentations, n (%)				
Occipital pain	22 (95.7)	12 (100)	10 (90.9)	0.48
Dizziness	3 (13.0)	2 (16.7)	1 (9.1)	1.00
Neurological deficit	1 (4.3)	1 (8.3)	0 (0.0)	1.00
Initial VW-MRI findings, n (%)				
Intimal flap	9 (39.1)	6 (50)	3 (27.3)	0.40
Intramural hematoma	21 (91.3)	11 (91.7)	10 (90.9)	1.00
Morphologic change				
Steno-occlusion	12 (52.2)	7 (58.3)	5 (45.5)	0.54
Dilatation	4 (17.4)	2 (16.7)	2 (18.2)	1.00
Pearl and string sign	3 (13.0)	2 (16.7)	1 (9.1)	1.00
Wall enhancement	23 (100)	12 (100)	11 (100)	1.00

Bold text indicates *P* value < 0.10

IQR, Interquartile range; M, Male; F, Female; VW-MRI, vessel wall magnetic resonance imaging

### Primary outcomes

**Fig. 2:**
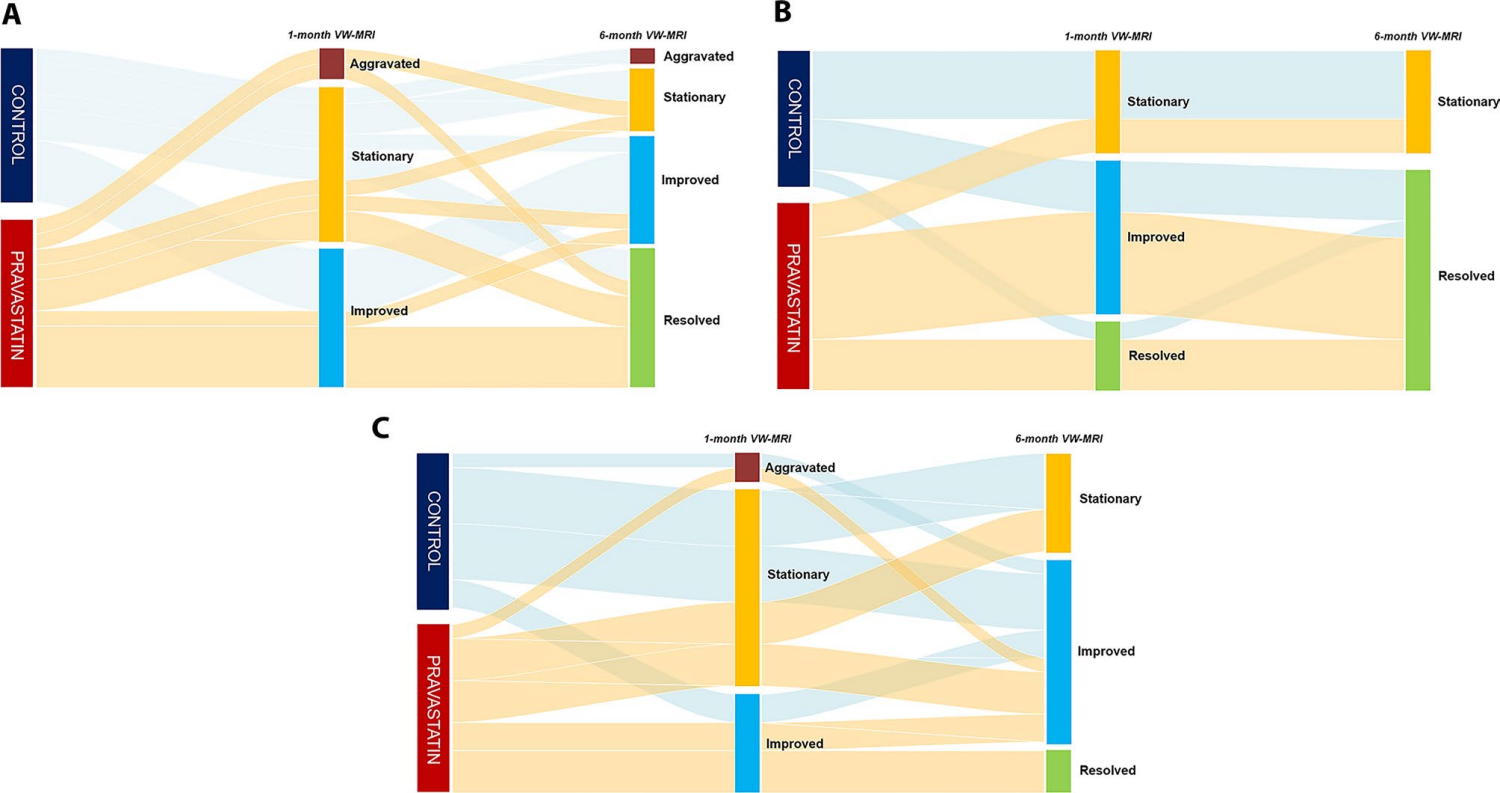
Alluvial diagram depicting the serial VW-MRI findings of intramural hematoma (**A**), vessel morphology (**B**) and wall enhancement (**C**) over the follow-up period. VW-MRI, Vessel wall magnetic resonance imaging

The primary outcomes with the VW-MRI findings in the early and late periods, are described in Table 2. In the early period, improved/resolved morphologic changes were identified more frequently in the pravastatin group than in the control group, with a borderline significance (81.8% versus 62.5%, *P* = .07). The other radiologic parameters were not significant between the two groups. In the late period, there were no differences in any VW-MRI findings between the two groups. However, improved/resolved states tended to be a little higher in the pravastatin group than in the control group (81.8% versus 50.0%, *P* = .32) despite the statistical insignificance. Alluvial diagram depicted the changing patterns of intramural hematoma, morphologic changes and wall enhancement in each group in Fig. 2A-C.

**Table 2:** Primary outcomes

	1-month VW-MRI Findings			6-month VW-MRI Findings		
	Pravastatin Group	Control Group	*P* value	Pravastatin Group	Control Group	*P* value
Intimal flap			1.00			1.00
Aggravated/stationary	1 (16.7)	1 (33.3)		1 (16.7)	0 (0.0)	
Improved/resolved	5 (83.3)	2 (66.7)		5 (83.3)	3 (100.0)	
Intramural hematoma			0.84			0.64
Aggravated/stationary	6 (54.5)	5 (50.0)		2 (18.2)	3 (30.0)	
Improved/resolved	5 (45.5)	5 (50.0)		9 (81.8)	7 (70.0)	
Morphologic change			**0.07**			0.32
Aggravated/stationary	2 (18.2)	5 (62.5)		2 (18.2)	4 (50.0)	
Improved/resolved	9 (81.8)	3 (37.5)		9 (81.8)	4 (50.0)	
Wall enhancement			0.37			0.67
Aggravated/stationary	7 (58.3)	9 (81.8)		3 (33.3)	4 (36.4)	
Improved/resolved	5 (41.7)	2 (18.2)		9 (66.7)	7 (63.6)	

Bold text indicates *P* value < 0.10

VW-MRI, vessel wall magnetic resonance imaging

In unadjusted analysis, age was the only significant variable of improved/resolved morphologic changes (crude OR: 0.813, 95% CI: 0.680-0.971, *P* = .02; Table 3). The use of pravastatin showed borderline significance for improved/resolved morphology in the acute period (crude OR: 7.500, 95% CI: 0.921-64.047, *P* = .06). In age-adjusted analysis, however, there was no independent risk factor.

**Table 3:** Age-adjusted analysis for prognostic factor of improved/resolved morphologic change in the early period (0–1 month)

	Crude OR (95% CI)	*P* value	Adjusted OR (95% CI)	*P* value
Age	0.813 (0.680-0.971)	**0.02**	NA	
Female	0.536 (0.081-3.533)	0.52	1.061 (0.078-14.494)	0.97
Hypertension	0.375 (0.055-2.555)	0.32	0.435 (0.036-5.278)	0.51
Smoking history	2.000 (0.166-24.069)	0.59	1.935 (0.083-45.333)	0.68
Occipital pain	NA		NA	
Neurologic deficit	0.500 (0.054-4.672)	0.54	0.078 (0.002-3.110)	0.18
Pravastatin use	7.500 (0.921-64.047)	**0.06**	0.853 (0.033-22.020)	0.92

Bold text indicates *P* value < 0.10

OR, odds ratio; CI, confidence interval; NA, not applicable

### Secondary outcomes

Clinically, all relevant dizziness in 3 patients and a neurological deficit presenting as a hemiparesthesia in 1 patient were resolved within 1 month. Most of the patients with sudden occipital pain were recovered at 1-month follow-up (95.5%) and completely resolved within 6 months. The recovery pattern was so favorable that there was no aggravation of the occipital pain in any patients. Also, there were no newly developed neurologic deficits.

The data of hs-CRP was partially obtained due to an equipment issue (*n* = 17). In the periodic laboratory examinations (Table 4), the pravastatin group showed that hs-CRP level decreased in a concave pattern in which its level decreased during the early period (0.070 to 0.045 mg/dL, Z score: -1.843, *P* = .07) and changed to plateau during the late period (0.045 to 0.050 mg/dL, Z score: − 0.985, *P* = .33). Meanwhile, the control group demonstrated the sharp increase in the hs-CRP level 3 times higher than the baseline during the early period (0.040 to 0.140 mg/dL, Z score: -2.371, *P* = .02) and the decrease on to the initial level during the late period. Significant decrease in LDL-C level up to 30% was observed in the pravastatin group in the early period (118.5 to 82.0 mg/dL, Z score: -2.276, *P* = .02).

**Table 4:** Periodic change in the high-sensitivity C-reactive protein (hs-CRP) and low-density lipoprotein (LDL-C)

		Median values (IQR)			In the early period (0–1 month)		In the late period (1–6 months)	
		Initially	In 1 month	In 6 months	Z-score	*P* value	Z-score	*P* value
hs-CRP^*^	Pravastatin group	0.070 (0.028-0.093)	0.045 (0.038-0.053)	0.050 (0.035-0.10)	-1.843	0.07	− 0.985	0.33
	Control group	0.040 (0.040-0.110)	0.140 (0.050-0.250)	0.040 (0.030-0.10)	-2.371	**0.02**	-2.207	**0.03**
LDL-C^†^	Pravastatin group	118.5 (83.5–142.0)	82.0 (63.75–111.0)	100.0 (68.3–100.0)	-2.276	**0.02**	-1.295	0.20
	Control group	132.0 (110.0-137.0)	129.0 (106.0-153.0)	146.0 (107.0-181.0)	− 0.445	0.66	− 0.356	0.72

Statistical analysis was performed using Wilcoxon signed-rank test

Bold text indicates *P* value < 0.10

^*^The included data was obtained due to an equipment issue (*n* = 17)

^†^The included data was derived from the both institutions (*n* = 23)

### Safety outcomes

All the patients accomplished 100% of the drug compliance, except one patient in the control who did not take aspirin for 1 month during the study period. (Supplementary Table S1). During the follow-up, there were no statin-related adverse events in terms of clinical and laboratory perspectives.

One patient (No.22) who had been allocated to the control group revisited the emergency department due to the repeat headache in 15 days after the initial symptoms. The angiographic study revealed the progression in extent and dilatation of the VAD. According to the study protocol, the patient was dropped out from the study. The patient prescribed a statin and an improved/resolved pattern was confirmed in 1-month follow-up angiographic study.

## Discussion

The present study was the first exploratory study pertaining to the pleiotropic effects of statin on the acute non-hemorrhagic VAD. In the pravastatin group, improved/resolved morphologic change was more observed with a borderline significance in the early period (within 1-month). Simultaneously, the level of hs-CRP was significantly decreased in the pravastatin group and vice versa in the control in the early period. There was no safety issue related to the pravastatin usage.

Since the discovery of statins in 1976 [32], they have been reported to have pleiotropic effects such as inflammation modulation in various clinical situations [33–36], in addition to their lipid-lowering role. In particular, the modulation of vascular inflammation and platelets activity through the inhibition of isoprenylation of signaling molecules by statins theoretically supports their effects not only in atherosclerosis but also in cervical artery dissection [37, 38]. The stabilization of vascular endothelial cells by statins has been reported in several in-vitro and animal studies. Landmesser et al. showed an increased endothelium resistance to oxidative stress and an enhanced activity of endothelial progenitor cells in the statin group, despite the similar decrease of LDL-C level [18]. Consequently, an increase of circulating endothelial progenitor cells are thought to contribute to angiogenesis, proliferation and regeneration of tissue-resident endothelium and restoration of barrier integrity [39]. Also, Walter et al. designed an animal study that simvastatin and saline injection were prepared to a balloon-injured rat arterial model. Re-endothelialization was accelerated in the peritoneal statin injection rat group, moreover, the acceleration rate was dose-dependent [20]. 

In this background, a lot of clinicians are using statins in acute VAD management. However, the clinical evidences for their use are scarce and the present study aimed to demonstrate the effect of statins on VAD for the first time through a randomized prospective exploratory study. Although the present study could not reveal the concrete conclusion due to in-nature limitation, we presented that pravastatin 40 mg had a potential effect for improved/resolved morphologic change of acute VAD in early period. As mentioned earlier, these outcomes can be attributed to the inflammation modulation by statins, and the reduction of hs-CRP in the pravastatin group supports this notion. In acute VAD, its morphologic changes are a common occurrence [40]. However, it is known that if the pattern progresses in an aggravating fashion, it could increase the risk of hemorrhagic and ischemic strokes. The present study has opened up the possibility of pravastatin’s favorable influence in the course of acute VAD, thereby cautiously inferring that it may ultimately lead to the reduction of VAD-related stroke. Despite the limited sample size of 23 participants, precluding the attainment of statistically significant outcomes, based on the results of this study, when comparison of proportions of 6-month morphologic changes in the pravastatin and control group was conducted, it is estimated that conducting an randomized controlled study with at least 34 patients in each group would be sufficient to demonstrate a statistically significant morphologic change between the pravastatin group and the control group.

There was no newly developed statin-related adverse event in this study. Of the statin-related adverse events, myopathy and diabetes mellitus were the well-proven ones [41]. Myopathy was known as the most common adverse effects of statin therapy. As the risk had a dose-dependency [42, 43], the standard dose, pravastatin 40 mg, might contribute to the satisfactory safety outcomes. Also, hydrophilicity of pravastatin might contribute no newly development of glucose intolerance or diabetes mellitus. Since statin uptake was initiated via a selective carrier-mediated transportation in liver, extra-hepatocyte diffusion was less in hydrophilic statins, which was associated with insulin sensitivity [44]. Even, previous systematic review presented pravastatin increased insulin sensitivity [45]. Therefore, the usage of pravastatin 40 mg seems to be acceptable, which would provide the dose reference for the future studies for the patients with non-hemorrhage VAD.

### Strength and limitations

Our study has some limitations. In the absence of randomized controlled trial on VAD as a reference, the sample size was determined by the annual patient count in each institution. The unanticipated COVID-19 pandemic significantly complicated recruitment. In spite of the randomization, it is thought that we failed to completely balance the baseline data between two groups because initial target number of patients to enroll was not achieved. Moreover, due to equipment issue, the data of hs-CRP was partially obtained. It hindered a comprehensive analysis using the marker level. We removed the parameter of inflammatory marker elevation from the prognostic factor analysis and just presented each level at different time points. Consequently, the study groups were not equivalent, consequently, the authors were unable to control potential confounding variables, such as age, which could have an impact on endothelium regeneration. The limited number of population and event made us only to present the study results with number and percentage, and simple comparison without a comprehensive analysis. Nevertheless, the present study had a potential benefits. First, we assessed radiologic, clinical and laboratory findings after dichotomization of study period. It enabled to present the changes at each stage as well as the comparison of the pattern. These assessment help to present the periodic variations despite the benign progression of non-hemorrhagic VAD. Also, up to date, the pleiotropic effects of statin for VAD was lay on theoretical or experimental perspectives. Previous reports already indicated that alterations in vascular condition were noted after three months in cases of vertebral artery dissection, with established knowledge that such changes may persist for up to six months [46, 47]. We first presented the potential benefit of statin for non-hemorrhagic VAD in the clinical setting, with consideration of VAD healing time scale. Therefore, the study results and included variables may serve as the foundation for future research regarding the calculation of sample size and study design. Taken together, future study should be designed to include statin effect on each step or mechanism of endothelium healing process in both experimental and clinical setting.

## Conclusion

The present exploratory randomized and controlled study showed that the pravastatin had a borderline effect on the morphologic recovery of acute-phase non-hemorrhage VAD. Therefore, future studies, including large sample sized prospective study and in-vitro laboratory examination, would be strongly required to elucidate the pleiotropic effects of statins on the non-hemorrhagic VAD.

## Electronic supplementary material

Below is the link to the electronic supplementary material.

Supplementary Material 1

## Data Availability

No datasets were generated or analysed during the current study.

## Abbreviations

VAD: Vertebral artery dissection
LDL-C: Low-density lipoprotein cholesterol
hs-CRP: High sensitivity C-reactive protein
