# Supplementary material for: Preliminary results of pravastatin on non-hemorrhagic vertebral artery dissection: an exploratory randomized and controlled study
Source: BMC Neurol. 2025 Jun 3;25:241. doi: 10.1186/s12883-025-04257-7 (PMC12131487; doi:10.1186/s12883-025-04257-7)
Supplement: Supplementary file 1 — Supplementary Material 1 [file 12883_2025_4257_MOESM1_ESM.pdf]

Supplementary method 1. Study eligibility criteria

Inclusion criteria
● Patients over 20 years old and under 75 years old<br>● In the last 1 month, mildly and medically controlled symptoms occurred, excluding hemorrhage and cerebral infarction of a moderate degree or severe, and unruptured vertebral artery dissection related to symptoms is radiologically identified<br>● Vertebral artery dissection in V4 segments (intradural location)<br>● Patients who agreed with this study
Exclusion criteria
● Vertebral artery dissection with hemorrhagic or moderate to severe ischemic cerebral infarction (National Institutes of Health Stroke Scale ≥ 5)<br>● Diabetes or other vascular endothelial abnormalities<br>● Currently taking statin or lipid lowering drugs for dyslipidemia or cardiovascular disease<br>● Previous endovascular treatment or surgery or radiotherapy in or near the lesion<br>● Active infection or severe trauma<br>● Poor medication compliance<br>● Past history of pravastatin, aspirin-induced hypersensitivity and adverse events<br>● Galactose/lactose intolerance, glucose-galactose malabsorption and genetic problem with pravastatin<br>● Severe liver failure or renal failure (eGFR < 30 ml/min/1.7 m2)<br>● History of myopathy including rhabdomyolysis<br>● Pregnant or possibly pregnant or who are lactating<br>● Heavy drinker<br>● Bleeding tendency or treated with peptic ulcer<br>● Patients taking medication which is contraindication of concurrent medication drugs with pravastatin, aspirin or has drug-drug interaction with pravastatin, aspirin

Supplementary method 2. Details of imaging parameters

TR, repetition time; TE, echo time; FOV, field-of-view

1. The pre- and post-contrast 3D T1-weighted images were obtained using following parameters:

- Discovery MR750w: TR, 591-650 ms; TE, 29.3-29.6 ms; FOV, 180-199 × 180-199 mm; matrix size, 260-292 × 260-292 mm and slice thickness, 0.6-0.8 mm.
- Ingenia CX: TR, 650 ms; TE, 34.8 ms; FOV, 180 × 180 mm; matrix size, 304 × 304 mm and slice thickness, 0.6 mm (interpolated to 0.3 mm).

2. The 3D proton density images were obtained using following parameters:

- Discovery MR750w: TR, 1800-2000 ms; TE, 36.8-40.2 ms; FOV, 180 × 180 mm; matrix size, 300-360 × 300-360 mm and slice thickness, 0.5-0.8 mm
- Ingenia CX: TR, 1,800 ms; TE, 37.4 ms; FOV, 180 × 180 mm; matrix size, 384 × 384 mm and slice thickness, 0.5 mm (interpolated to 0.3 mm)

3. The time-of-flight images were obtained using following parameters:

- Discovery MR750w: TR 20-23 ms; TE 3.4-3.7 ms; FOV, 210-240 × 210-240 mm, matrix size, 448-536 × 256-378 mm and slice thickness, 1.2-1.4mm.
- Ingenia CX: TR,19 ms; TE, 3.5 ms; FOV, 210 × 210 mm; matrix size, 640 × 330 mm and slice thickness, 1.2 mm (interpolated to 0.6 mm)

Supplementary table 1. Adherence to medication

	Pravastatin (n=12)	Control (n=11)
Took 100% of study drug	12 (100%)	10 (90.9%)
Took ≥ 80% of study drug	12 (100%)	11 (100%)
